# The Facts about Food after Cancer Diagnosis: A Systematic Review of Prospective Cohort Studies

**DOI:** 10.3390/nu12082345

**Published:** 2020-08-05

**Authors:** Emanuele Rinninella, Maria Cristina Mele, Marco Cintoni, Pauline Raoul, Gianluca Ianiro, Lucia Salerno, Carmelo Pozzo, Emilio Bria, Maurizio Muscaritoli, Alessio Molfino, Antonio Gasbarrini

**Affiliations:** 1 UOC di Nutrizione Clinica, Dipartimento di Scienze Mediche e Chirurgiche, Fondazione Policlinico Universitario A. Gemelli IRCCS, Largo A. Gemelli 8, 00168 Rome, Italy; 2 Dipartimento di Medicina e Chirurgia Traslazionale, Università Cattolica del Sacro Cuore, Largo F. Vito 1, 00168 Rome, Italy; mariacristina.mele@unicatt.it (M.C.M.); emilio.bria@unicatt.it (E.B.); antonio.gasbarrini@unicatt.it (A.G.); 3 UOSD di Nutrizione Avanzata in Oncologia, Dipartimento di Scienze Mediche e Chirurgiche, Fondazione Policlinico Universitario A. Gemelli IRCCS, Largo A. Gemelli 8, 00168 Rome, Italy; pauline.raoul1@gmail.com; 4 Scuola di Specializzazione in Scienza dell’Alimentazione, Università di Roma Tor Vergata, Via Montpellier 1, 00133 Rome, Italy; marco.cintoni@gmail.com; 5 UOC di Medicina Interna e Gastroenterologia, Dipartimento di Scienze Mediche e Chirurgiche, Fondazione Policlinico Universitario A. Gemelli IRCCS, Largo A. Gemelli 8, 00168 Rome, Italy; gianluca.ianiro@hotmail.it; 6 Freelance Registered Dietitian; luciasalerno.dietista@gmail.com; 7 Comprehensive Cancer Center, Fondazione Policlinico Universitario A. Gemelli IRCCS, Largo A. Gemelli 8, 00168 Rome, Italy; carmelo.pozzo@policlinicogemelli.it; 8 Dipartimento di Medicina Traslazionale e di Precisione, Università degli Studi di Roma “La Sapienza”, Piazzale Aldo Moro 5, 00185 Roma, Italy; maurizio.muscaritoli@uniroma1.it (M.M.); alessio.molfino@uniroma1.it (A.M.)

**Keywords:** cancer, diet, malnutrition, meat, milk, dairy products, nuts, vegetarian diet, ketogenic diet, mediterranean diet, gut microbiota, short chain fatty acids, personalized medicine

## Abstract

Nutritional guidelines suggest specific energy and protein requirements for patients with cancer. However, cancer patients, often malnourished, use self-made or web-based diets to ameliorate the prognosis of their disease. This review aimed to investigate the associations between post-diagnostic diet and prognostic outcomes in cancer patients. A systematic literature search was performed in Pubmed and Web of Science databases from inception to 30 October 2019, based on fixed inclusion and exclusion criteria. The risk of bias was assessed. A total of 29 prospective studies was identified. Breast (*n* = 11), colorectal (*n* = 9), prostate (*n* = 8) cancers are the most studied. Low- fat diet, healthy quality diet, regular consumption of fiber such as vegetables and high-quality protein intake are beneficial while Western diet (WD) and high consumption of saturated fats could be associated with a higher risk of mortality. Bladder (*n* = 1), gynecological (*n* = 1), lung, stomach, and pancreatic cancers still remain almost unexplored. This systematic review suggested that detrimental dietary patterns such as WD should be avoided but none of the food categories (meat, dairy products) should be eliminated in cancer patients’ diet. Further large prospective studies are needed to assess the role of post-diagnostic diet in patients with cancer.

## 1. Introduction

According to the World Health Organization (WHO), the cancer burden rose to 18.1 million new cases and 9.6 million cancer deaths in 2018 [1]. Convincing evidence supports a reduced risk of different types of cancer among healthy populations that follow specific dietary regimens [2,3,4]. Indeed, a recent meta-analysis suggested an association between healthy dietary patterns and decreased risk of colon and breast cancer [2]. Especially, adherence to the Mediterranean diet is associated with a lower incidence of several cancer types such as colorectal cancer [3]. The beneficial effects of Mediterranean diet are mainly driven by higher intakes of fruit, vegetables, and whole grains [4]. In contrast, evidence for the role of post-diagnostic diet in cancer survival remains limited. However, malnutrition, defined as a state resulting from lack of intake of nutrition that leads to altered body composition, is common among cancer patients due to the disease itself and oncologic treatments [5,6]. To prevent malnutrition, energy and protein requirements for cancer patients are largely widespread by international guidelines [7,8,9] but little is known about the food choices and dietary regimen a cancer patient should benefit from. In this context, cancer patients are often motivated to learn how food choices and dietary patterns can improve their nutritional status and response to treatment and reduce risk of cancer recurrence and cancer-specific mortality. Thus, web sources are full of fake prescriptions or confounding statements easily shared by patients without any endorsement by the scientific community [10]. Many “cancer diets” are often restrictive, avoiding a whole nutrient class (i.e., meat or dairy products) in the misleading belief that certain foods “feed the tumor” [10]. In the last decades, a growing number of prospective cohort studies [11,12,13,14,15,16] have investigated the association between post-diagnostic dietary patterns—such as Western diet (WD) and prudent diet (PD)—or food type—such as meat, dairy products, dietary fiber, nuts—and prognostic outcomes among patients with different cancers. This systematic review aims to investigate possible associations between dietary patterns/choices after cancer diagnosis and prognostic outcomes (i.e., mortality, cancer progression, and recurrence) in patients affected by main solid tumors.

## 2. Methods

This systematic review was performed according to the Cochrane Handbook for systematic reviews [17] and followed the Preferred Reporting Items for Systematic Reviews and Meta-Analyses (PRISMA) statement [18].

### 2.1. Eligibility Criteria

Inclusion criteria were:

- Population: adult (≥18 years old) patients diagnosed with breast, gastrointestinal (gastric, pancreatic, colorectal), gynecological (uterine, cervical, ovarian, endometrial, vulvar), lung and urological (prostate, bladder) cancers.
- Exposure: any post-diagnostic dietary exposure such as dietary patterns or individual food components exposure (fruit, vegetables, dairy, meat, fish, cereals) or use of diet quality indices.
- Study design: prospective or retrospective cohort studies.
- Outcomes of interest: overall survival (OS) or all-cause mortality (ACM), cancer-specific mortality (CSM), death from a cause other than specific cancer, cancer progression, disease-free survival (DFS), cancer recurrence and recurrence-free survival (RFS).

Exclusion criteria were studies including patients with other types of cancer; reviews, comments, editorials, case series, or meeting abstracts.

### 2.2. Definitions of Outcomes

OS and ACM were defined as the time from cancer diagnosis to death from any cause. CSM was defined as the time from specific cancer diagnosis to death from this specific cancer. DFS was defined as the time from cancer diagnosis to tumor recurrence or death from any cause. RFS was defined as the time from the cancer diagnosis to tumor recurrence.

### 2.3. Data Sources and Search Strategy

The search was carried out on 30th October 2019 using two electronic databases, MEDLINE (via PubMed) and ISI Web of Science. The search strategy was limited to English language articles and there were no restrictions on the date of publication. The search string for each database is described in Table S1 (Supplementary File). The reference lists of retrieved articles were manually scrutinized to identify potentially relevant studies.

### 2.4. Study Selection

The study selection process was independently carried out by three reviewers (P.R.; E.R.; L.S.). All articles generated from the electronic search were imported into Mendeley© (Elsevier, Amsterdam, The Netherlands) a references’ management software, and duplicates were removed. Titles and abstracts of all records were screened for eligibility based on inclusion criteria. All titles assessed as ineligible were excluded. Differences in judgment during the selection process between the three reviewers were settled by discussion and consensus.

### 2.5. Data Extraction

Information was collected using an Excel© (Microsoft Office, Redmond, WA, USA) spreadsheet specifically developed for this study. Each full-text article was retrieved, and the articles deemed ineligible were excluded and the reasoning reported. Differences in judgment among reviewers were settled by discussion and consensus.

### 2.6. Quality Assessment

Two reviewers independently assessed the risk of bias of each included study using the Quality In Prognosis Studies (QUIPS) tool as described by Hayden et al. [19]. The QUIPS tool developed to assess the risk of bias in prognostic factor studies has six domains: study participation, study attrition, prognostic factor measurement, outcome measurement, study confounding, and statistical analysis and reporting. Each of the six domains was rated as having a ‘Low’, ‘Moderate’, or ‘High’ risk of bias. Subsequently, the overall risk of bias was established for each study. The overall risk of bias was considered low if ≤2 domains were rated a moderate risk of bias and all others were rated a low risk of bias. The overall risk of bias was considered moderate if >2 domains were rated a moderate risk of bias and all others were rated a low risk of bias. The overall risk of bias was considered high if ≥1 domain was rated the high risk of bias, irrespective of all other domains. Differences in judgment among reviewers were settled by discussion and consensus.

### 2.7. Data Synthesis

Because of the high heterogeneity of the studies, a systematic review was performed. Indeed, the dietary assessments, dietary factors/dietary patterns, and outcomes of each study were not comparable and consequently, a meta-analysis was unfeasible. The main results of the review were displayed on a summary of findings table. For each study, first author’s last name, year of publication, country of origin, types of outcome, sample size of the population, period time of diagnosis, mean follow-up duration, exposure assessment, diet-quality indices, dietary patterns or other dietary factors, adjustment covariates, and multivariate-adjusted risk estimates—hazards ratios (HR) or risk ratios (RR) for the highest vs. the lowest category with their corresponding 95% confidence intervals (CI) were reported.

## 3. Results

### 3.1. Study Selection

**Figure 1:**
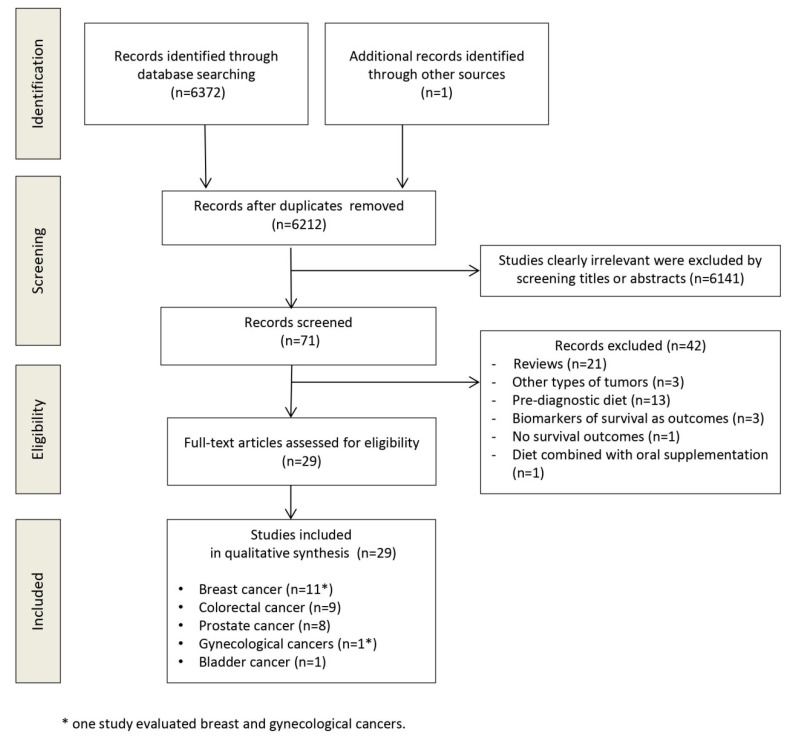
Preferred reporting items for systematic reviews and meta-analyses (PRISMA) flow diagram.

The flow diagram in Figure 1 displays the results of the literature search and study selection process. A total of 6372 publications were initially identified, 161 were duplicates. Hand searching allowed the identification of one additional study. Twenty-nine studies were identified for inclusion in the systematic review.

### 3.2. Study Characteristics

Table 1 shows the main characteristics of the included studies [11,12,13,14,15,16,20,21,22,23,24,25,26,27,28,29,30,31,32,33,34,35,36,37,38,39,40,41,42]. All included studies are prospective. Eleven studies enrolled patients with breast cancer [10,19,20,21,22,23,24,25,26,27,28], 9 with colorectal cancer [11,12,29,30,31,32,33,34,35], 8 with prostate cancer [13,35,36,37,38,39,40,41], 1 with bladder cancer [16] and 1 with gynecological cancer [29] (ovarian, uterine and cervical cancers). The sample size ranged from 230 [29] to 9514 [26] patients. Twenty-three studies were performed in the USA, 1 in Japan [34], 1 in China [23], 1 in China and USA [26], 1 in the United Kingdom [16], 1 in Germany [15] and 1 in Denmark [28]. The median duration of follow-up ranged from 2 [14] to 28 years [40]. Eight [12,15,21,22,25,29,31,41] out of 29 studies assessed the influence of dietary patterns while all other studies assessed the effect of dietary factors. Twenty studies evaluated ACM, 17 CSM, 8 cancer recurrence, 4 cancer progression, 4 death from non-specific cancer cause, 3 OS, 3 DFS, and 3 RFS. Studies evaluated different dietary patterns, different diet quality indices, or individual dietary factors. The definitions of the dietary patterns and diet quality indices are detailed in Table S2 (Supplementary File).

### 3.3. Quality Assessment

According to the QUIPS tool, 13 [11,12,21,24,25,26,27,31,33,35,37,39,41] studies had a low overall risk of bias, 14 studies a moderate overall risk of bias [13,14,15,20,22,26,28,30,32,34,36,38,40,42] and 2 studies a high overall risk of bias [16,29]. The overall risk of bias of each study is presented in Table 2 and the risk of bias of each item is detailed in Table S3 (Supplementary file).

Fourteen studies provided an adequate description of the source population, baseline study sample, recruitment, and inclusion/exclusion criteria for participants. For two studies [16,29], the risk of bias of study population was high due to a too small sample size. For fourteen studies [13,14,15,20,22,23,28,30,32,34,36,38,40,42], the risk of bias of study population had a moderate risk of bias mainly due to the missing of tumor stage data. Regarding study attrition, all studies had a low risk of bias, providing adequate response rate for study participants, adequate description of attempts to collect information on participants who dropped out and reasons for loss to follow-up. Regarding prognostic factor assessment, all studies had a moderate risk of bias. Indeed, included studies used either food frequency questionnaire (FFQ) or 24-h recall as a dietary assessment tool, providing recall bias in dietary intake assessment. In fact, FFQ or 24-h recall provided measurement errors mainly due to a reporting average intake over a long period of time with under- or over-estimated intake. For the majority of studies, the definitions of outcomes were clear and the methods of outcome measurement were valid and reliable. However, for five studies [21,22,25,31,36], despite a thorough review of records by physicians, some causes of death could have been misclassified. Regarding study confounding, the majority of studies (*n* = 19) had a moderate risk of bias. Indeed, some studies did not consider the pre-diagnostic diet as confounding factor [14,22,33,38,40,42], or treatment information on 25% of the cohort was missing [30], a too small number of confounders were considered [29,32,34,36], or inconsistent patterns of lifestyle factors between participants lowered the statistical power of the study [26]. Finally, three studies [23,28,29] had a moderate risk of bias in reporting of results.

### 3.4. Summary of Findings

#### 3.4.1. Breast Cancer

Table 2 reports the statistically significant results (*p*-value < 0.05), HR or RR with 95% CI, of the included studies enrolling patients diagnosed with breast cancer.

##### Dietary Patterns

A cohort study [25] of 2729 women from the Nurses’ Health Study with invasive stage I–III breast cancer found, in simple and multivariate-adjusted analyses, no association of CSM with the post- diagnostic Modified Mediterranean Diet Score (MMDS). However, in a multivariate analysis, a higher MMDS was significantly associated with a lower risk of non-breast cancer death in breast cancer women with low physical activity (adjusted RR, 0.39, 95% CI 0.20–0.75) [25].

A recent study [29] of 110 breast cancer women found no significant association between Mediterranean Diet Score (MDS) and ACM. However, a higher Healthy Eating Index (HEI) score (≥70) was significantly associated with lower ACM (adjusted HR 0.49 95% CI 0.25–0.97) [29].

Two cohort studies of 2619 [21] and 1901 [22] breast cancer women assessed after diagnosis, WD and Prudent Diet (PD) in multivariate-adjusted analyses. A significant association between higher adherence to PD and lower risk of ACM (adjusted HR 0.57 95% CI 0.36–0.90) was found in one study [25]. As regards WD, no association was found with ACM or CSM in both studies. Moreover, both studies found a significant association between death from causes other than breast cancer and PD (respective adjusted RR: Q5 vs. Q1 0.54 95% CI 0.31–0.95 [21]; Q4 vs. Q1 HR 0.35 95% CI 0.17–0.73 [22]). In contrast, Kroenke et al. found a significant association between WD and this outcome (Q5 vs. Q1 adjusted RR 2.31, 95% CI 1.23–4.32) [21].

##### Dietary Factors

A large multi-center prospective cohort study including 4441 breast cancer women [24], after adjustment for factors at diagnosis, suggested that higher intake of saturated (Q5 vs. Q1 adjusted HR 1.41 95% CI 1.06–1.87) and trans fat (Q5 vs. Q1 adjusted HR 1.78 95% CI 1.35–2.32) in the post- diagnostic diet was significantly associated with a higher risk of ACM [24]. No significant association was observed between CSM and the respective intake of meat and dairy products.

A large prospective cohort study included 1982 women with diagnosed breast cancer and followed for 18 years: in multivariate analyses of diet after diagnosis, no significant association was found between low-fat and high-fat dairy intake and ACM [20]. Another large prospective cohort study [27] enrolling 1893 women diagnosed with early-stage invasive breast cancer suggested that, compared with the reference (0 to <0.5 servings/day), those consuming larger amounts of high-fat dairy (≥1.0 servings/day) had a higher risk of ACM (adjusted HR 1.64 95% CI 1.24–2.17) and death from non-breast cancer causes (adjusted HR 1.67 95% CI 1.13–2.47). Recently, a prospective cohort Danish study [28] suggested no associations between post-diagnostic intake of total dairy products (milk yogurt and cheese) and cancer recurrence, CSM, and ACM [28].

A large prospective study of more than 6000 breast cancer patients [11] observed a relationship between higher intake of protein and reduced risk of recurrence (Q4 vs. Q1 adjusted RR 0.75 95% CI 0.61–0.91), which was particularly significant for protein from animal sources (Q4 vs. Q1 adjusted RR 0.78 95% CI 0.63–0.95). Moreover, higher animal protein intake was correlated with a lower risk of CSM (Q4 vs. Q1 adjusted RR 0.77 95% CI 0.62–0.94) [11]. These findings confirmed the results of a large cohort study [20] of 1982 breast cancer women showing a significant association between ACM and total protein intake (Q5 vs. Q1 adjusted RR 0.65 95% CI 0.47–0.88).

In a large study of combined data on 9514 US and Chinese breast cancer women [26], post- diagnosis soy food consumption corresponding to about 10 mg isoflavones/day was associated with a non-significant reduced risk of CSM but a statistically significant reduced risk of disease recurrence (adjusted HR 0.75 95% CI 0.61–0.92). Another cohort study [23] of 5042 female Chinese breast cancer survivors showed that soy food intake, as measured by either soy protein or soy isoflavone intake, was inversely associated with ACM (Q4 vs. Q1 adjusted HR 0.67 95% CI 0.51–0.88) and recurrence (Q4 vs. Q1 adjusted HR 0.66 95% CI 0.52–0.84) [23].

Three studies [20,24,28] investigated post-diagnostic intake of whole-grain products and breast cancer prognosis, neither reporting any associations. However, specifically, Andersen et al. [32] found a significant association between post-diagnostic serving size increment of rye bread per day and higher risk of CSM (adjusted HR 1.29 95% CI 1.02–1.63), hypothetically due to the habit of the Danish population to eat rye bread with high-fat products such as butter, cheese and processed meat [28].

#### 3.4.2. Colorectal Cancer

Table 3 reports the statistically significant results (*p*-value < 0.05), HR or RR with 95% CI, of the included studies enrolling patients diagnosed with colorectal cancer.

##### Dietary Patterns

One prospective cohort of 1009 stage III colorectal cancer patients [12] reported significant worse OS (adjusted HR 2.32 95% CI 1.36–3.96), DFS (adjusted HR 3.25 95% CI 2.04–5.19) and RFS (adjusted HR 2.85 95% CI 1.75–4.63) in the highest quintile of WD compared to lowest one. Another cohort study [31] of 1201 colorectal cancer patients diagnosed with stage I–III colorectal did not show significant associations between WD and survival. Regarding PD, both studies found no significant association with prognostic endpoints.

One German study [15] of 1404 colorectal cancer patients evaluated MMDs and the Healthy Nordic diet index (HNDI). Higher MMDS was associated with lower ACM (adjusted HR 0.48 95% CI 0.32–0.74).

##### Dietary Factors

A recent study [33] examined marine ω-3 polyunsaturated fatty acids (PUFAs) and fish intake and survival endpoints among 1011 colorectal cancer patients enrolled in Cancer and Leukemia Group B (CALGB) 89,803 (Alliance) trial (an adjuvant chemotherapy trial) and found that high intake of marine ω-3 PUFAs was associated with longer DFS (adjusted HR 0.72 95% CI 0.54–0.97). Moreover, colorectal cancer patients who consumed dark fish ≥1/week experienced longer DFS (adjusted HR 0.65 95% CI 0.48–0.87), RFS (adjusted HR 0.61 95% CI 0.46–0.86), and OS (adjusted HR 0.68 95% CI 0.48–0.96) [33].

Regarding fiber intake, a Japan cohort study [34] of 5864 colorectal cancer patients demonstrated that a low consumption of green leafy vegetables was correlated with worse ACM (never-consumers vs. everyday consumers, adjusted HR 1.87 95% CI 1.22–2.88). Another cohort of 1575 colorectal cancer patients [35] analyzed total fiber, cereal fiber, vegetable fiber, fruit fiber, and whole-grain intakes after diagnosis. A 5 g/day increment of total fiber and cereal fiber significantly decreased ACM (adjusted HRs total fiber: 0.86 95% CI 0.65–0.93, cereal fiber: 0.78, 95% CI 0.68–0.90) and CSM (adjusted HRs total fiber: 0.78 95% CI 0.65–0.93, cereal fiber 0.67 95% CI 0.50–0.90) [42]. Moreover, a 5 g/day increment of vegetable fiber significantly correlated with ACM (adjusted HR 0.83 95% CI 0.72–0.96) and 20 g/day of whole-grain intake significantly correlated with CSM (adjusted HR 0.72 95% CI 0.59–0.88) [35].

Two observational cohort studies of 1201 [31] and 2315 [30] non-metastatic colorectal cancer patients did not observe any association between red and processed meat intake after diagnosis and both ACM and CSM. However, colorectal cancer patients with consistently high red and processed meat intake before and after diagnosis had a higher risk of CSM (RR 1.79 95% CI 1.11–2.89) compared with those with consistently low intake [30]. Moreover, the above-mentioned Japanese study [40] did not show any significant association between meat and ACM.

One study [32] evaluated associations of dairy product intakes after diagnosis with ACM and CSM among 1111 colorectal cancer patients. An inverse association with ACM was observed for post- diagnosis milk intake (RR 0.72 95% CI 0.55–0.94) [32].

The study of Fung et al. [31] of 1201 women diagnosed with stage I–III colorectal cancer found a higher ACM risk with the consumption of sugar-sweetened beverages and fruit juices (for each additional serving/day, adjusted HR 1.11 95% CI 1.01–1.23).

In a study [13] examining 826 patients with stage III colon cancer enrolled in the CALGB 89,803 trial, a higher nut intake (≥2 servings/week of total nut intake vs. never) was associated with a significant improvement in OS (adjusted HR 0.43 95% CI 0.25–0.74) and DFS (adjusted HR 0.58 95% CI 0.37–0.92). Moreover, a subgroup analysis revealed that higher tree nut intake (≥2 servings/week of total nuts intake vs. never) significantly improved OS (adjusted HR 0.47 95% CI 0.27–0.82) and DFS (adjusted HR 0.54 95% CI 0.34–0.85) [13]. Interestingly, the above-mentioned study of Fung et al. [31] found a significantly lower risk of CSM for each serving/day of nuts consumption (adjusted HR 0.69 95% CI 0.49–0.97).

#### 3.4.3. Prostate Cancer

Table 4 reports the statistically significant results (*p*-value < 0.05), HR or RR with 95% CI, of the included studies enrolling patients diagnosed with prostate cancer.

##### Dietary Patterns

One prospective study examined post-diagnostic Mediterranean diet adherence in relation to CSM and ACM among 4538 men initially diagnosed with prostate cancer (clinical stage T1–T3a) [41]. Lower ACM was associated with greater adherence to the Mediterranean diet after diagnosis (high adherence vs. low adherence adjusted HR 0.78 95% CI 0.67–0.90) [41].

Regarding fat intake after diagnosis, a prospective study of 4577 men with non-metastatic prostate cancer suggested that higher ACM was associated with saturated fats (Q5 vs. Q1 adjusted HR 1.30 95% CI 1.05–1.60) and trans fats (Q5 vs. Q1 adjusted HR 1.25 95% CI 1.05–1.49) intakes after diagnosis [39]. Another study [42] on 926 men with non-metastatic prostate cancer confirmed the adverse effect of saturated fats on ACM and showed that men who obtained more of their daily calories from vegetable fats and less of their daily calories from carbohydrates had a 33% lower risk of ACM (adjusted HR 0.67 95% CI 0.47–0.96) [42].

##### Dietary Factors

Two studies of 1294 [14] and 1202 men with prostate cancer [36] assessed the association between the consumption of processed red meat (salami, sausage, bacon and hot dogs) after diagnosis and prostate cancer progression; both showed a non-significant increase in the risk of disease progression (*p* > 0.05) [14,36]. As regards poultry, one study [14] reported that consuming poultry after diagnosis is not significantly associated with prostate cancer progression. However, men who reported consuming higher amounts of poultry with skin after prostate cancer diagnosis had a significantly higher risk of prostate cancer progression (Q3 vs. Q1 adjusted HR 2.26 95% CI 1.36–3.76) [14].

A prospective study [37] including 3918 men with prostate cancer showed that total milk and dairy intakes after diagnosis were not associated with a greater risk of CSM at multivariate analysis. However, men with the highest versus lowest intake of whole milk had increased risk of cancer progression (Q5 vs. Q1 adjusted HR 2.15 95% CI 1.28–3.60) [37]. Song et al. [40] confirmed that higher whole milk intake was associated with worse cancer progression (>2.5 servings/day vs. ≤0.5 servings/day, adjusted HR 2.17 95% CI 1.34–3.51).

As regards fiber intake, a cohort study of 1560 non-metastatic patients with prostate cancer [38] observed that patients consuming ≥5.7 servings/day of cruciferous vegetables after diagnosis—such as broccoli, cauliflower, cabbage, brussels sprouts—had 59% lower risk of cancer progression compared to patients consuming ≤1.4 servings/day of these vegetables (adjusted HR 0.41 95% CI 0.22–0.76). No significant association was found between prostate cancer progression and other vegetables and fruit consumption [38].

Interestingly, a study of 1202 men with non-metastatic prostate cancer in the Health Professionals’ Follow-up Study (HPFS) [36] examined the influence of the post-diagnostic intake of tomato products on prostate cancer progression, suggesting a beneficial effect of tomato sauce consumption (Q1 vs. Q4 adjusted HR 0.56 95% CI 0.38–0.82). However, a study by Richman et al. [38] found no significant associations with prostate cancer progression and tomato sauce.

#### 3.4.4. Bladder Cancer

One recent prospective study [16] of 389 patients with bladder cancer investigated the influence of post-diagnostic vegetables and fruit intake on cancer recurrence. No significant association was found between fruit and vegetable intakes and bladder cancer recurrence (*p* > 0.05). According to the QUIPS tool, the overall risk of bias in this study is high.

#### 3.4.5. Gynecological Cancers

A recent study [29] of 120 gynecological cancer women (i.e., ovarian (*n* = 19), cervical (*n* = 54), and uterine cancer (*n* = 47)) investigated the association of post-diagnostic diet quality assessed by HEI and MDS with ACM. A high HEI score (≥70) was associated with lower mortality (adjusted HR 0.92 95% CI 0.89–0.96). No significant association between MDS and ACM was found. According to the QUIPS tool, the overall risk of bias in this study is high.

#### 3.4.6. Lung, Gastric and Pancreatic Cancers

We found no data showing an association between post-diagnostic food choices/dietary patterns and survival outcomes in patients diagnosed with lung, gastric, and pancreatic cancers.

## 4. Discussion

The overall results of this systematic review highlight that none of the food categories should be eliminated by cancer patients. Especially, there is no clear association between consumption of meat or animal products and cancer progression/recurrence or CSM after a cancer diagnosis. However, a significant positive association between detrimental dietary patterns such as WD and cancer progression was found. On the contrary, high consumption of fiber such as whole grain cereals, green and cruciferous vegetables seems to be protective against cancer progression and mortality.

Breast, colorectal and prostate cancers are the most studied. In patients with breast cancer, PD after diagnosis could improve OS. PD is characterized by a diet high in fruits, vegetables, whole grains, legumes, poultry, fish and low-fat products. This finding is consistent with results of studies assessing a positive effect of pre-diagnostic components of PD on breast cancer prognosis. Indeed, previous studies found that, before diagnosis, whole grain [43,44], fruit and vegetable [45] intakes could be associated with a lower risk of mortality after breast cancer. Several studies suggested that a high fiber intake was associated with a reduction in circulating estrogen and androstenedione levels which are involved in the pathogenesis of breast cancer [46,47]. Indeed, fiber may bind estrogens in the colon during the enterohepatic circulation and increase the fecal excretion of estrogens [44]. On the other hand, WD and higher intake of saturated fats/trans after diagnosis could increase ACM, particularly deaths from other causes than breast cancer. WD, based on high intake of refined grains, processed and red meats, desserts, fats, and sweets, is characterized by a high glycemic load which may promote weight gain and an increment of fat mass [48]. Weight gain and increase of fat mass with a concurrent loss in muscle mass have shown to worsen clinical outcomes of breast cancer treatments [24]. Moreover, our results showed that higher intake of protein after diagnosis could be associated with reduced risk of breast cancer recurrence [11,20]. Particularly, in more than 6000 breast cancer patients, protein intake from animal sources (meat, poultry, fish, dairy products, and eggs) could be associated with lower risk of CSM [11,20]. Considering that increased protein intake contributes to greater strength and muscle mass gains when combined with physical exercise [49], higher intake of proteins (particularly animal proteins) with physical activity after diagnosis could counterbalance loss of muscle mass known to jeopardize survival of several cancers [50,51] including breast cancer. However, post-diagnostic consumption of high-fat dairy products could increase ACM and death from other causes than breast cancer [27] whereas total dairy products and low-fat dairy products were not associated with breast cancer prognostic outcomes [20,28]. We can hypothesize that estrogenic hormones found in dairy fat may be detrimental to breast cancer survival [27]. To date, even if further studies are needed to clarify mechanisms of all these findings, clinicians and breast cancer patients can be reassured that consuming animal protein-containing foods is not likely to increase CSM and ACM.

As regards colorectal cancer, high adherence to healthy diet and Mediterranean diet, in particular MMDS for Americans, could decrease cancer mortality and recurrence. Several clinical studies have reported an association of the components of Mediterranean diet—such as olive oil polyphenols and tomato lycopene—with a reduction in cancer initiation and progression [52]. Moreover, Mediterranean diet has been shown to be rich in antioxidants which may be associated with lower plasma concentrations of inflammatory markers [53]. Furthermore, an increment of dietary fiber from vegetables and whole grain after colorectal diagnosis could significantly improve ACM and CSM [34,35]. We can hypothesize that various components in whole grain and vegetables such as phytoestrogens, vitamins, antioxidants and microelements have beneficial influence on colorectal cancer. Moreover, dietary fiber derived from whole grain, fruits and vegetables could lower ACM and CSM after breast cancer by adjusting the gut microbiota and metabolism [54,55]. Indeed, short chain fatty acids (SCFAs) are the end-products of fiber and complex carbohydrates fermentation of most of the bacterial strains in the colon [56]. Several studies [57,58,59] showed a strong association between gut microbiota composition and cancer. Recently, a prospective cohort study [60] showed a significant association between higher concentrations of SCFAs and longer PFS in cancer patients. Additionally, our results suggested that a high consumption of nuts could improve OS and DFS in patients with colorectal cancer. Indeed, nuts—rich in unsaturated fatty acids, fiber, minerals, phenolic antioxidants, and phytosterols—could inhibit colorectal cancer growth by inducing cell cycle arrest, apoptosis, and by inhibiting cell proliferation, angiogenesis, and metastasis [61,62]. Moreover, consumption of marine ω3 PUFAs and dark fish (such as salmon particularly high in marine ω-3 PUFAs) improves DFS and OS [33]. Indeed, in vitro, studies showed that ω3 PUFAs have antiproliferative and apoptotic effects on human colorectal cancer stem-like cells [63,64]. On the other hand, high adherence to WD after diagnosis could worsen OS, DFS, and RFS in colorectal cancer. The study of Fung et al. [65] showed that WD is positively correlated with levels of biomarkers of obesity and cardiovascular disease risk such as serum insulin [65]. Both insulin and insulin like growth factors have been shown to be associated with enhanced tumor growth and antiapoptosis [66]. Hence, WD may increase the risk of cancer progression and recurrence. Notwithstanding red and processed meat intake may increase the risk of developing colorectal cancer [67,68], no significant association was found between mortality and post-diagnostic meat and red meat consumption [30,31,34]. Moreover, a reduction of meat consumption might result in loss of muscle mass which could worsen clinical outcomes and increase risk of colorectal cancer mortality. However, long-term meat consumption may be a more relevant measure and may be better represented by pre-diagnostic diet than by post- diagnostic diet [30].

Regarding prostate cancer, adherence to the Mediterranean diet could decrease ACM [41]. Mediterranean diet is mainly characterized by the consumption of olive oil. Animal and in vitro model studies [69,70] showed that polyphenols found in vegetable fats—such as hydroxytyrosol in olive oil—could induce apoptosis and suppress multiple oncogenic signaling pathways in prostate cancer cells. Also, tomato is a main component of Mediterranean diet, however, a positive association between tomato sauce consumption and prostate cancer prognosis remains under debate [36,38]. Lycopene, a carotenoid present in tomatoes, could inhibit the proliferation of androgen-dependent human prostate tumor cells through specific molecular pathways [71]. Further research on the role of tomatoes after prostate cancer diagnosis is needed. In addition, Richman et al. suggested that patients consuming cruciferous vegetables after prostate cancer diagnosis—such as broccoli, cauliflower, cabbage, brussels sprouts—had lower risk of cancer progression [38]. Cruciferous vegetables are composed of phytochemicals such as glucosinolates. Glucosinolates are hydrolyzed to form isothiocyanates and indoles, which have anticarcinogenic effects in vitro and in vivo [72]. Nevertheless, because of the small sample size of the study of Richman et al. [38] and the lack of assessment of pre-diagnostic diet, these results should be taken with caution. Vegetable fat intake after diagnosis was associated with a lower risk of CSM and ACM [39]. Indeed, animal and in vitro data suggest that components in olive oil inhibit migration, invasion, and adhesion of prostate cancer cells [70], and walnuts reduce prostate tumor growth [73] and inhibit androgen receptor expression in prostate cancer cells [74]. On the contrary, post-diagnostic intake of saturated and trans fat could increase the risk of ACM [39,42]. This could be explained by several biological mechanisms. High-fat diet including saturated and trans fats could modulate androgen signaling [75], upregulate the insulin-like growth factor pathway and increase prostate inflammation, thereby promoting prostate cancer growth [76]. Saturated and trans-fatty acid intake also may raise serum cholesterol levels [77,78]. Cholesterol is required for cell growth and may promote prostate cancer development [79]. Hence, perturbations of cholesterol homeostasis could enhance prostate cancer progression and mortality [79]. Like cholesterol, choline is highly concentrated in prostate cancer cells, and blood concentrations of choline have been associated with an increased risk of prostate cancer [80]. Eggs, milk, and poultry are the main sources of choline. This could explain that high consumption of eggs, poultry with skin, and whole milk could increase cancer progression and ACM. However, in a prospective cohort study of 47,896 men [81] post-diagnostic choline intake was not significantly associated with mortality. Further studies are needed to examine the relation of dietary choline to choline concentrations in the prostate and the effects of dietary choline in malignant prostate cells.

For bladder and gynecological cancers, poor data was available, and the results of the studies did not show any association of food components or diet quality scores [16,29]. Post-diagnostic diet of lung and stomach cancer patients still remains totally unexplored. More research is required to propose an adequate nutritional support to these patients to improve their nutritional status and prognosis [82,83]. Regarding pancreatic cancer, despite exclusion criteria (diet combined with supplementation [84], animal model [85]), few studies [84,85] could not be ignored. In vitro, mice models have shown significantly lower pancreatic tumor weight and higher muscle weight in patients fed with a ketogenic diet (KD) versus a standard diet [85]. KD could be a potential chemotherapy adjuvant in pancreatic cancer patients reducing glucose uptake, necessary for cancer cell cycle [86]. However, the effects of a long-term KD on health and microbiota remain controversial [56]. Consequently, further studies with larger sample size are required to assess the potential effect of KD as adjuvant therapy on patients’ survival and pancreatic tumor progression. A recent study [84] investigated the effects of alkalization diet (with supplementary oral sodium bicarbonate) during chemotherapy on the survival of 28 advanced pancreatic patients. The alkaline diet consists of a daily intake of at least 400 g of fruits and vegetables and no intake of meat and dairy products. From the start of alkalization therapy, the median OS of the patients with high urine pH (>7.0) was significantly longer than those with a low urine pH (≤7.0) [84]. Vegetables and fruits intake may modify tumor metabolism through a reduction of the inflammation, a moderate caloric restriction, and inducing changes in patient’s insulin levels and gut microbiome [84]. However, since the sample size of this study is very small, further studies are needed to confirm these interesting results.

This systematic review has several strengths. The majority of the included studies are cohort studies with large sample size, long follow-up durations, and low to moderate risk of bias. However, some limitations could not be ignored. First, data from the studies, prospective in nature, are observational; therefore, the possibility of residual confounding and causal interference cannot be excluded. Secondly, there is a high heterogeneity between studies due to variations in dietary exposure, dietary assessment methods, and population characteristics, making meta-analyses unfeasible. Third, information on the tumor stage and histology was unfortunately not available in several included studies. Furthermore, in most studies, the exposure to dietary patterns or factors was assessed by FFQs, consequently, errors in measurement need to be considered. Indeed, an imprecise measurement of consumption might have attenuated the true associations. Moreover, most studies did not assess pre-diagnostic dietary data, however, assessing the changes from pre- diagnostic to post-diagnostic diet would be relevant because the association observed after diagnosis may reflect intake before diagnosis.

## 5. Conclusions

Detrimental dietary patterns such as WD and the high consumption of some food categories (saturated/trans fats, high-fat dairy products) could worsen prognostic outcomes in breast, colorectal and prostate cancer patients. Nevertheless, animal proteins such as fish, poultry, low-fat dairy products and meat should not be excluded from cancer patient’s diet. More research is needed to confirm these findings and better clarify the impact of diet after diagnosis in cancer patients. Further investigation is warranted to explore the role of post-diagnostic diet in the most common cancers such as lung, stomach, gynecological, bladder, and pancreatic cancer.

## Figures and Tables

**Table 1:** Main characteristics of the included prospective studies.

First Author, Year	Country	Type of Cancer	Years of Diagnosis	N° of Cases (% of Cases Completed Follow-Up)	Cancer Stage at Diagnosis	Median Duration of Follow-Up	Type of Diet/Food Evaluated	Outcomes
Holmes, 1999 [20]	USA	Breast	1976–1990	1982 (95)	NR	18 years	Fat Protein Red meat Fiber Poultry, Dairy Others 80 food items	ACM
Kroenke, 2005 [21]	USA	Breast	1982–1998	2619 (84.2)	I–III	9 years	PD WD	ACM CSM Death from non-breast cancer causes
Kwan, 2009 [22]	USA	Breast	1997–2000	1901 (88.1)	I–III	5.9 years	PD WD	ACM CSM Death from non-breast cancer causes Recurrence
Shu, 2009 [23]	China	Breast	2002–2006	5042 (91.2)	I–IV	3.9 years	Soy food	ACM Cancer recurrence CSM
Beasley, 2011 [24]	USA	Breast	1987–1999	4441 (96.9)	I–III	5.5 years	Monounsaturated fats Polyunsaturated fat Saturated fats Trans fats Carbohydrates Protein	ACM CSM
Kim, 2011 [25]	USA	Breast	1978–1998	2729 (79.0)	I–III	NR	Diet quality scores	ACM CSM Death from non-breast cancer causes
Nechuta, 2012 [26]	USA China	Breast	1991–2006	9514 (90.7)	I–III	7.4 years	Soy food	ACM CSM Recurrence
Kroenke, 2013 [27]	USA	Breast	1997–2000	1893 (80.3)	I–IIIa	11.8 years	Total dairy High-fat dairy Low-fat dairy	ACM CSM Recurrence Death from non-breast cancer causes
Holmes, 2017 [11]	USA	Breast	1976–2004	6348 (70.9)	I–III	NR	Total protein Vegetable protein Animal protein Red meat Poultry Fish High-fat dairy Low-fat dairy	CSM Recurrence
Andersen, 2019 [28]	Denmark	Breast	1993–1997	1965 (76.6)	NR	7 years	Total whole grain products Whole grain bread Rye bread Oatmeal/muesli Total dairy products Milk Yogurt Cheese	ACM CSM Recurrence
Karavasiloglou, 2019 [29]	USA	Breast and gynecological	1988–1994	230 (110 breast/120 gynecological)	survivors	16 years	Mediterranean diet Healthy American diet	ACM
Meyerhardt, 2007 [12]	USA	Colorectal	1999–2001	1009 (75.1)	III	5.3 years	PD WD	OS DFS RFS
Mc Cullough, 2013 [30]	USA	Colorectal	1992–2003	2315 (58.3)	NR	4.6 years	Red and processed meat	ACM CSM
Fung, 2014 [31]	USA	Colorectal	1986–2008	1201 (63.8)	I–III	11.2 years	PD WD AHEI-2010 components	ACM CSM
Yang, 2014 [32]	USA	Colorectal	1992–2009	1.111 (14.5)	NR	7.5 years	Milk	ACM CSM
Van Blarigan, 2018 [33]	USA	Colon	1999–2001	1011 (69.8)	III	7 years	Dark fish Marine ω-3 PUFA	OS DFS RFS
Ratjen, 2017 [15]	Germany	Colorectal	2004–2007	1404 (85.5)	NR	7 years	Modified Mediterranean diet Healthy Nordic diet	ACM
Tamakoshi, 2017 [34]	Japan	Colorectal	2003–2008	5864 (91.1)	NR	7.4 years	Green leafy vegetables Meat	ACM
Fadelu, 2018 [13]	USA	Colon	1999–2001	826	III	6.5 years	Total nuts Tree nuts Peanuts	OS DFS RFS
Song, 2018 [35]	USA	Colorectal	1980–2010	1575 (50.9)	I–III	8 years	Total fiber Cereal fiber Vegetable fiber Fruit fiber Whole grain	ACM CSM
Chan, 2006 [36]	USA	Prostate	1986–1996	1202 (NR)	I–III	77 months ± 34	Red meat Grains Vegetables, Fruit Milk, Tomatoes Tomato sauce Fish	Cancer progression
Richman, 2010 [14]	USA	Prostate	2004–2005	1294 (90.2)	NR	2 years	Red processed and unprocessed red meat Fish Poultry Eggs	Cancer recurrence or progression
Petterson, 2012 [37]	USA	Prostate	1986–2006	3918 (94.2)	I–III	7.6 years	Skim and low-fat milk Whole milk Total milk low-fat Dairy low-fat products Dairy full-fat products Total dairy products	ACM CSM
Richman, 2012 [38]	USA	Prostate	2000–2003	1560 (NR)	I–III	23 months	Total vegetables Cruciferous vegetables Tomato sauce Legumes Other vegetable sub-groups Total fruit Subgroups of fruit	Cancer progression
Richman, 2013 [39]	USA	Prostate	1986–2010	4577 (76.8)	I–III	8.4 years	Saturated, monounsaturated, polyunsaturated, trans, animal, and vegetable fat	ACM CSM
Song, 2013 [40]	USA	Prostate	1982–2010	2806 (89.1)	NR	28 years	Different milk types	Cancer progression
Kenfield, 2014 [41]	USA	Prostate	1986–2010	4538 (74.0)	I–III	8.9 years	Mediterranean diet	ACM CSM
Van Blarigan, 2015 [42]	USA	Prostate	1982–1997	926 (64.0)	I–III	10 years	Saturated fat Monounsaturated, Polyunsaturated, Trans fats Animal fat Vegetable fat Carbohydrates	ACM CSM
Joechems, 2018 [16]	UK	Bladder	2005–2011	389	I–III	3.7 years	Fruit Vegetables	Cancer recurrence

Abbreviations: ACM, all-cause mortality; AHEI, alternate healthy eating index, CSM, cancer-specific mortality; NR, not reported; OS, overall survival; PD, prudent diet; PFS, progression-free survival; PUFA, polyunsaturated fatty acid; RFS, recurrence-free survival; WD, western diet.

**Table 2:** Summary of results of the included studies enrolling patients diagnosed with breast cancer. Statistically significant (*p* < 0.05) results, HR or RR with 95% CI, are reported.

Study ID	Method of Dietary Assessment	Time of Dietary Assessment	Dietary Factor or Dietary Patterns Evaluated	Outcomes	HR or RR (95% CI)	Adjustment Covariates	QUIPS Score
Holmes, 1999 [20]	FFQ	After diagnosis	Fat Protein Red meat Fiber Poultry, dairy Others 80 food items	ACM	Protein intake Q5 vs. Q1: RR 0.65 (0.47–0.88)	Age at diagnosis, year of diagnosis, tumor size, grade, hormone receptor status, and the presence of positive lymph nodes, menopausal status, family history of breast carcinoma, age at first pregnancy, parity, postmenopausal hormone use, oral contraceptive use, and BMI	Moderate
Kroenke, 2005 [21]	FFQ	More than 1 year after diagnosis	PD WD	ACM CSM Death from non-breast cancer causes	No significant results No significant results PD Q5 vs. Q1: RR 0.54 (0.31–0.95) WD Q5 vs. Q1: RR 2.31 (1.23–4.32)	Age, BMI, oral contraceptive use, menopausal status, age at menopause, use of postmenopausal hormone therapy, breast cancer stage using the standard American Joint Committee on Cancer staging criteria, chemotherapy, and hormonal therapy, energy intake and alcohol intake	Low
Kwan, 2009 [22]	FFQ	After diagnosis	PD WD	ACM Death from non-breast cancer causes CSM Recurrence	PD Q4 vs. Q1: HR 0.57 (0.36–0.90) PD Q4 vs. Q1: HR 0.35 (0.17–0.73) No significant results No significant results	Age at diagnosis and total energy intake, total physical activity at baseline, BMI at enrollment and smoking status	Moderate
Shu, 2009 [23]	Dietary questionnaire	6 months after diagnosis	Soy food	ACM Cancer recurrence CSM	Soy food Q4 vs. Q1: HR 0.67 (0.51–0.88) Soy food Q4 vs. Q1: HR 0.66 (0.52–0.84) No significant results	Age at diagnosis, TNM stage, chemotherapy, radiotherapy, type of surgery received, BMI, ER and PR status, tamoxifen use, education level, crucifer intake, red meat intake, fish intake, any vitamin supplement use, tea consumption and physical activity	Moderate
Beasley, 2011 [24]	FFQ	After diagnosis	Monounsaturated fats PUFAs Saturated fats Trans fats Carbohydrates Protein	ACM CSM	Saturated fats Q5 vs. Q1: HR 1.41 (1.06–1.87) Trans fats Q5 vs. Q1: HR 1.78 (1.35–2.32) No significant results	Age, state of residence, menopausal status, smoking, breast cancer stage, alcohol, history of hormone replacement therapy), interval between diagnosis and diet assessment, and at follow-up (energy intake, breast cancer treatment, body mass index, and physical activity	Low
Kim, 2011 [25]	FFQ	After diagnosis	Diet quality	ACM CSM Death from non-breast cancer causes	No significant results No significant results MMDS Q3 vs. Q1: RR 0.39 (0.20–0.75) (with low physical activity ≤9 METs/week)	Age, energy intake, disease stage (I, II, III), treatment, smoking status, physical activity, menopausal status and hormone therapy use, oral contraceptive use, BMI, weight change, energy intake, multivitamin use, alcohol	Low
Nechuta, 2012 [26]	FFQ 24-h dietary recall	After diagnosis	Soy food	ACM CSM Recurrence	No significant results No significant results Consumption of ≥10 mg soy isoflavones/d: HR 0.75 (0.61–0.92)	Age, TNM stage, chemotherapy, radiotherapy, hormonal therapy, education, race, ethnicity, first-degree family history of breast cancer, menopausal status, parity, recreational physical activity in metabolic equivalent hours per week, smoking, cruciferous vegetable intake, and BMI	Low
Kroenke, 2013 [27]	FFQ	After diagnosis	Total dairy High-fat dairy Low-fat dairy	ACM Death from non-breast cancer causes CSM Recurrence	High-fat dairy: ≥1 serving/day vs. 0 to <0.5 serving/day: HR 1.64 (1.24–2.17) High-fat dairy: ≥1 serving/day vs. 0 to <0.5 serving/day: HR 1.67 (1.13–2.47) No significant results No significant results	Age, dairy intake and breast cancer outcomes, stage, tumor size, grade, nodal status, estrogen receptor status, human epidermal growth factor receptor 2 status, treatment, education, ethnicity, energy intake, red meat, fiber, and fruit intake, BMI, physical activity, alcohol intake, smoking status	Low
Holmes, 2017 [11]	FFQ	At least 12 months after diagnosis	Total protein Vegetable protein Animal protein Red meat Poultry Fish High-fat dairy Low-fat dairy	CSM Recurrence	Animal protein Q4 vs. Q1: RR 0.77 (0.62–0.94) Total protein Q4 vs. Q1: RR 0.75 (0.61–0.91) Animal protein Q5 vs. Q1: RR 0.78 (0.63–0.95)	Age, energy intake, BMI, weight change, menopausal status, hormone therapy use, age at first birth, parity, alcohol consumption, aspirin use, oral contraceptive use, year of diagnosis, disease stage, self-reported radiation therapy, chemotherapy, and hormonal treatment, smoking, physical activity	Low
Andersen, 2019 [28]	FFQ	After diagnosis	Total whole grain products Whole grain bread Rye bread Oatmeal/muesli Total dairy products Milk Yogurt Cheese	ACM CSM Recurrence	No significant results Rye bread: by serving size increment per day HR 1.29 (1.02–1.63) No significant results	Age at diagnosis, educational level, physical activity, BMI, smoking, alcohol intake, tumor stage, number of affected lymph nodes, ER status, year of diagnosis	Moderate
Karavasiloglou, 2019 [29]	24-h dietary recall interview	After diagnosis	Diet quality (HEI) Mediterranean diet (MDS)	ACM	HEI score (≥70 vs. <70): HR 0.49 (0.25–0.97)	Age, ethnicity, time between cancer diagnosis and completion of the NHANES III questionnaire, socioeconomic status, marital status, BMI, physical activity, self-reported prevalent chronic diseases at baseline, daily energy intake and history of menopausal hormone therapy use	High

Abbreviations: ACM, all-cause mortality; BMI, body mass index; ChT, chemotherapy, CI, confidence interval; CSM, cancer-specific survival; DQIR, Quality Index-Revised; ER, estrogen receptor; FFQ, food frequency questionnaire; HEI, Healthy eating index; HR, hazard ratio; MDS, Mediterranean Diet Score; MET, Metabolic Equivalent of Task; MMDS, Modified Mediterranean Diet Score; *p*, *p*-value; PD, prudent diet; PR, progesterone receptor; Q1, lowest tertile or quartile or quintile; Q3, highest tertile; Q4, highest quartile; Q5, highest quintile; QUIPS, quality assessment of prognosis cohort studies; RFS, Recommended Food Score; RR, risk ratio; SFFQ, semi-quantitative food frequency questionnaire; TNM, Tumour Node Metastasis; vs, versus, WD, Western diet.

**Table 3:** Summary of results of included studies regarding colorectal cancer. Statistically significant (*p* < 0.05) results (HR or RR; 95% CI) are reported.

Study ID	Method of Dietary Assessment	Time of Dietary Assessment	Dietary Factor or Dietary Patterns Evaluated	Outcomes	HR or RR (95% CI)	Adjustment Covariates	QUIPS Score
Meyerhardt, 2007 [12]	SFFQ	In the middle of ChT course and approx. 6 months after ChT	WD PD	OS DFS RFS	WD Q5 vs. Q1: HR 2.32 (1.36–3.96) WD Q5 vs. Q1: HR 3.25 (2.04–5.19) WD Q5 vs. Q1: HR 2.85 (1.75–4.63) No significant results	Sex, age, nodal stage, body mass index, physical activity level, baseline, performance status, or treatment group	Low
Mc Cullough, 2013 [30]	FFQ	After diagnosis	Red and processed meat	ACM CSM	No significant results	Age, sex, tumor stage, pre-diagnostic diet, race/ethnicity, education, smoking, history of hypertension, physical activity; alcohol intake; nonsteroidal anti-inflammatory drug use; multivitamin use; postmenopausal hormone use; family history of CRC; type of treatment; history of high cholesterol, stroke, or lung disease; total folate; dietary folate; total calcium; dietary calcium; and fruit, vegetables, whole grains, and fish/poultry consumption	Moderate
Fung, 2014 [31]	FFQ	at least 6 months after diagnosis	AHEI-2010 components MMDS DASH WD PD	ACM CSM	Sugar-sweetened beverages + juices for each additional serving: HR 1.11 (1.01–1.23) AHEI Q5 vs. Q1: HR 0.71 (0.52–0.98) Nuts for each serving/day: HR 0.69 (0.49–0.97)	Age, physical activity, BMI, weight change, cancer grade, chemotherapy, smoking status, energy intake, colon or rectal cancer, stage of disease, date of colorectal cancer diagnosis	Low
Yang, 2014 [32]	FFQ	After diagnosis	Milk intake	ACM	Milk Q4 vs. Q1: RR 0.72 (0.55–0.94)	Age at diagnosis, sex, tumor stage at diagnosis	Moderate
Van Blarigan, 2018 [33]	FFQ	During and 6 months after ChT	Dark fish Marine ω-3 PUFA	OS DFS RFS	Dark fish ≥1/week vs. never: HR 0.68 (0.48–0.96) Dark fish ≥1/week vs. never: HR 0.65 (0.48–0.87) Marine ω-3 PUFA Q4 vs. Q1: HR 0.72 (0.54–0.90) Dark fish ≥1/week vs. never: HR 0.61 (0.46–0.86)	Sex, energy intake, age, stage, number of positive lymph nodes, treatment arm, BMI, physical activity, smoking, and aspirin use	Low
Ratjen, 2017 [15]	SFFQ	Median of 6 years after diagnosis.	MMD Healthy Nordic diet	ACM	MMDS Q4 vs. Q1: HR 0.48 (0.32–0.74) No significant results	Sex, age, BMI, physical activity, survival time from CRC diagnosis, tumor location, occurrence of metastases, occurrence of other cancers, chemotherapy, smoking status and total energy intake	Moderate
Tamakoshi, 2017 [34]	Interview	After diagnosis	Green leafy vegetables Meat	ACM	Green leafy vegetables never consumers vs. everyday consumers: HR 1.87 (1.22–2.88) No significant results	Sex, institutions and adjusted for age and entry year	Moderate
Fadelu, 2018 [13]	FFQ	After diagnosis	Total nuts Tree nuts Peanuts	OS DFS RFS	Total nuts 0 vs. ≥2 servings/week: HR 0.43 (0.25–0.74) Tree nuts 0 vs. ≥2 servings/week: HR 0.47 (0.27–0.82) Total nuts 0 vs. ≥2 servings/week: HR 0.58 (0.37–0.92) Tree nuts 0 vs. ≥2 servings/week: HR 0.54 (0.34–0.85) No significant results	Calorie intake, age, sex, depth of invasion through bowel wall, number of positive lymph nodes, baseline performance status, treatment group, body mass index, physical activity, aspirin use, and glycemic load	Moderate
Song, 2018 [35]	FFQ	Between 6 months and 4 years after diagnosis	Total fiber Cereal fiber Vegetable fiber Fruit fiber Whole grain	ACM CSM	Total fiber 5 g/day increment: HR 0.86 (0.65–0.93) Cereal fiber 5 g/day increment: HR 0.78 (0.68–0.90) Vegetables fiber 5 g/day increment: HR 0.83 (0.72–0.96) Total fiber 5 g/day increment: HR 0.78 (0.65–0.93) Cereal fiber 5 g/day increment: HR 0.67 (0.50–0.90) Whole grain 20 g/day increment: HR 0.72 (0.59–0.88)	Age at diagnosis, sex, year of diagnosis, tumor stage, anatomic subsite, and differentiation, BMI, physical activity, alcohol consumption, aspirin use, vitamin D, total fat, folate, calcium, and glycemic load, pre-diagnostic diet	Low

Abbreviations: ACM, all-cause mortality; AHEI, Alternate Healthy Eating Index; BMI, body mass index; ChT, chemotherapy, CI, confidence interval; CRC, colorectal cancer, CSM, cancer-specific survival; DASH, Dietary Approaches to Stop Hypertension; FFQ, food frequency questionnaire; HNFI, Healthy Nordic Food Index; HR, hazard ratio; MDS, Mediterranean Diet Score; MMD, Modified Mediterranean Diet; MMDS, Modified Mediterranean Diet Score; NR, not reported; OS, overall survival; *p*, *p*-value; PD, prudent diet; PFS, progression-free survival; PUFA, polyunsaturated fatty acids; Q1, lowest quintile/quartile; Q4, highest quartile; Q5, highest quintile; QUIPS, quality assessment of prognosis cohort studies; RFS, recurrence-free survival; RR, risk ratio; SFFQ, semi-quantitative food frequency questionnaire; vs, versus; WD, Western diet.

**Table 4:** Summary of results of included studies regarding prostate cancer. Statistically significant (*p* < 0.05) results (HR or RR; 95% CI) are reported.

Study ID	Method of Dietary Assessment	Time of Dietary Assessment	Dietary Factor or Dietary Patterns Evaluated	Outcomes	HR or RR (95% CI)	Adjustment Covariates	QUIPS Score
Chan, 2006 [36]	SFFQ	After diagnosis	Red meat Grains Vegetables, Fruit Milk Tomatoes Tomato sauce Fish	Cancer progression	Tomato sauce Q4 vs. Q1: HR 0.56 (0.38–0.82)	Total energy, age, clinical factors, and pre-diagnostic diet	Moderate
Richman, 2010 [14]	FFQ	After diagnosis	Red processed and unprocessed meat Fish Poultry Eggs	Cancer progression or recurrence	Eggs Q4 vs. Q1: HR 2.02 (1.10, 3.72) Poultry with skin Q3 vs. Q1: HR 2.26 (1.36–3.76)	Age at diagnosis, energy intake (kcal/d), and time from diagnosis to questionnaire Other food groups, clinical T stage at diagnosis, smoking, race, education, income, marital status, vigorous activity, and frequency of fried food intake	Moderate
Petterson, 2012 [37]	FFQ	After diagnosis	Skim and low-fat milk Whole milk Total milk low-fat Dairy low-fat products Dairy full-fat products Total dairy products	CSM ACM	Whole milk Q5 vs. Q1: HR 2.15 (1.28–3.60) No significant results	Age at diagnosis, total caloric intake, smoking status, BMI, exercise, alpha-linolenic acid intake, TNM stage, Gleason score	Low
Richman, 2012 [38]	SFFQ	After diagnosis	Total vegetables Cruciferous vegetables Tomato sauce Legumes Other vegetable sub-groups Total fruit Subgroups of fruit	Cancer progression	Cruciferous vegetables Q4 vs. Q1: HR 0.41 (0.22–0.76)	Age, energy intake, prognostic risk at diagnosis, primary treatment, BMI, walking metabolic equivalent task, Gleason score, quartile ranks of eggs, poultry with skin, fruit, and vegetables other than the exposure of interest	Moderate
Richman, 2013 [39]	FFQ	After diagnosis	Saturated, monounsaturated, polyunsaturated, trans, animal, and vegetable fat	ACM CSM	Vegetable fats Q5 vs. Q1: HR 0.74 (0.61- 0.88) Saturated fats Q5 vs. Q1: HR 1.30 (1.05–1.60) Trans fats Q5 vs. Q1: HR 1.25 (1.05–1.49) Vegetable fats Q5 vs. Q1: HR 0.71 (0.51–0.98)	For CSM: age, energy intake, clinical-stage, BMI; vigorous activity, smoking, calcium, alcohol, protein, the other fats, pre-diagnostic intake of the exposure of interest For ACM: above covariates + parental history of myocardial infarction before age 60, high blood pressure at diagnosis, diabetes mellitus at diagnosis, elevated cholesterol at diagnosis, and presence of co-morbidities	Low
Song, 2013 [40]	FFQ	After diagnosis	Different types of dairy products	Cancer progression	Whole milk >2.5 servings/day vs. ≤0.5 servings/day: HR 2.17 (1.34–3.51)	Age, baseline, smoking, vigorous exercise, alcohol intake, race, diabetes status, red meat consumption, and assignment in the original trial	Moderate
Kenfield, 2014 [41]	FFQ MDS	After diagnosis	Mediterranean diet	ACM	MDS high vs. low: HR 0.78 (0.67–0.90)	Age, period time, energy, BMI, vigorous physical activity, smoking status, prostate-specific antigen screening history	Low
Van Blarigan, 2015 [42]	FFQ	5 years after diagnosis	Saturated fat Monounsaturated, Polyunsaturated, Trans fats Animal fat Vegetable fat Carbohydrates	ACM CSM	5% more of their daily calories from saturated fat and 5% less of their daily calories from carbohydrate: HR 1.81 (1.20–2.74) 10% more of their daily calories from vegetable fats and 10% less of their daily calories from carbohydrates: HR 0.67 (0.47–0.96) 5% more of their daily calories from saturated fat and 5% less of their daily calories from carbohydrate: HR 2.78 (1.01–7.64)	Age at diagnosis, caloric intake, modified D’Amico risk category, primary treatment, BMI, smoking, and intake of alcohol, protein, and other fats	Moderate

Abbreviations: ACM, all-cause mortality; BMI, body mass index; ChT, chemotherapy, CI, confidence interval; CSM, cancer-specific mortality; FFQ, food frequency questionnaire; HNFI, Healthy Nordic Food Index; HR, hazard ratio; MDS, Mediterranean Diet Score; *p*, *p*-value; Q1, lowest quantile; Q3, highest tertile; Q4, highest quartile; Q5, highest quintile; QUIPS, quality assessment of prognosis cohort studies; RR, risk ratio; SFFQ, semi-quantitative food frequency questionnaire; vs, versus.

## Supplementary Materials

The following are available online at https://www.mdpi.com/2072-6643/12/8/2345/s1, Table S1: Full search strategies for electronic databases; Table S2: Definitions of dietary patterns and diet quality scores; Table S3: Bias assessment results for each study and each domain using the Quality in Prognostic Studies (QUIPS) tool.
