# Supplementary material for: The Facts about Food after Cancer Diagnosis: A Systematic Review of Prospective Cohort Studies
Source: Nutrients. 2020 Aug 5;12(8):2345. doi: 10.3390/nu12082345 (PMC7468771; doi:10.3390/nu12082345)
Supplement: Supplementary file 1 [file nutrients-12-02345-s001.pdf]

Supplementary file

Table S1. Full search strategies for electronic databases.

1.1 Medline

Medline (via Pubmed) was searched for articles published through 30th October 2019

Set	Search terms	Search type	Results
1	diet OR dietary OR food OR foods OR beverage OR beverages OR bread OR cereals OR grains OR whole-grain OR soy OR soya OR potatoes OR legumes OR rice OR pasta OR vegetables OR fruit OR milk OR dairy OR eggs OR meat OR fish OR seafood OR nuts OR sweets OR alcohol OR coffee OR tea OR juice OR fats OR "fatty acids" OR carbohydrates OR fibre OR fiber OR sugar	Advanced	1,855,677
2	cancer OR tumor OR tumour OR neoplasms [MeSH]	Advanced	2,461,548
3	patients OR patient	Advanced	6,362,661
4	1 AND 2 AND 3	Advanced	33,026
5	4 NOT risk NOT parenteral NOT enteral NOT vitamin NOT mineral	Advanced	23,854
6	**Refined by:** clinical trials AND review AND English language AND humans	Advanced	4,911

1.2. Web of Science

Web of Science was searched for articles published through 30th October 2019

Set	Search terms	Search type	Results
1	**Title:** diet OR dietary OR food OR foods OR beverage OR beverages OR bread OR cereals OR grains OR whole-grain OR soy OR soya OR potatoes OR legumes OR rice OR pasta OR vegetables OR fruit OR milk OR dairy OR eggs OR meat OR fish OR seafood OR nuts OR sweets OR alcohol OR coffee OR tea OR juice OR fats OR carbohydrates OR fibre OR fiber OR sugar	Advanced	1,933,069
2	**Title:** cancer OR tumor OR tumour OR neoplasm	Advanced	1,696,108
3	**Title:** patients OR patient	Advanced	2,274,948
4	1 AND 2 AND 3	Advanced	1,461

Table S2. Definitions of dietary patterns and diet quality scores

**Definitions**	
**Dietary patterns**	
Prudent diet (PD)	PD is characterized by a diet high in fruits, vegetables, whole grains, legumes, poultry, fish, and low-fat dairy products.
Western diet (WD)	WD is characterized by high intake of refined grains, processed and red meats, high-fat dairy products, desserts, sweets, sweetened beverages, animal fat (such as butter), snacks and french fries.
Mediterranean diet (MD)	MD is characterized by a proportionally high consumption of olive oil, legumes, unrefined cereals, fruits, and vegetables, moderate to high consumption of fish, moderate consumption of dairy products, moderate wine consumption, and low consumption of non-fish meat products.
**Dietary quality scores**	
Healthy Eating Index (HEI)	HEI is a measure of diet quality, independent of quantity,that can be used to assess compliance with the U.S. Dietary Guidelines for Americans. The higher a participant's score on the HEI, the better the diet according to the Dietary Guidelines for Americans and the Food Guide Pyramid.
Alternate Healthy Eating Index-2010 (AHEI-2010)	Components for AHEI-2010 were chosen based on their association with chronic diseases shown in the literature. The AHEI-2010 awards points for higher consumption of vegetables (excluding potatoes), whole fruit, whole grains, nuts and legumes, long chain omega-3 fatty acids, polyunsaturated fat; a lower consumption of sugar-sweetened beverages, red/processed meat, sodium, trans fat, and moderate alcohol consumption.
Mediterranean diet score (MDS)	MDS assesses the adherence to the Mediterranean diet by including the consumption of 9 components: legumes, vegetables, fruit and nuts, cereals, fish and seafood, meat and meat products, dairy products, the ratio of monounsaturated to saturated fats and alcohol.
Modified Mediterranean Diet score (MMDS)	MMDS was adapted from the Trichopolou score for the American population. It awards 1 point for intake was greater than the cohort specific median in vegetables, legumes, fruits, nuts, whole grains, fish, and monounsaturated: saturated fat ratio; and one point if intake was less than the cohort median in meat, and if alcohol intake between 5 and 15 g/d for women. The MMDS was often used in American cohort studies because in non-Mediterranean countries polyunsaturated lipids are the principal unsaturated lipids in diet.

Dietary Approaches to Stop Hypertension (DASH) score	DASH score was developed based on foods that are emphasized and discouraged in the DASH trial which was originally designed for blood pressure reduction. The DASH diet aims to reduce the sodium in diet promoting a variety of foods rich in nutrients that help lower blood pressure, such as potassium, calcium and magnesium. These include whole grains, fruits, vegetables low-fat dairy products, some fish, poultry and legumes.
Healthy Nordic Diet Index (HNFI)	HNFI is based on six typical food groups consumed in the Nordic countries: fish, cabbage, apple and pears, root vegetables, rye bread and oatmeal.

Table S3. Bias assessment results for each study and each domain using the Quality in Prognostic Studies (QUIPS) tool.

First Author, Year	Study participation	Study attrition	Prognostic factor measurement	Outcome measurement	Study confounding	Statistical analysis and reporting	Overall risk of bias
Andersen, 2019	Moderate	Low	Moderate	Low	Low	Moderate	**Moderate**
Beasley, 2011	Low	Low	Moderate	Low	Moderate	Low	**Low**
Chan, 2006	Low	Low	Moderate	Moderate	Moderate	Low	**Moderate**
Fadelu, 2018	Moderate	Low	Moderate	Low	Moderate	Low	**Moderate**
Fung, 2014	Low	Low	Moderate	Moderate	Low	Low	**Low**
Holmes, 1999	Moderate	Low	Moderate	Low	Moderate	Low	**Moderate**
Holmes, 2017	Low	Low	Moderate	Low	Low	Low	**Low**
Joechems, 2018	High	Low	Moderate	Low	Low	Low	**High**
Karavasiloglou, 2019	High	Low	Moderate	Low	Moderate	Moderate	**High**
Kenfield, 2014	Low	Low	Moderate	Low	Moderate	Low	**Low**
Kim, 2011	Low	Low	Moderate	Moderate	Low	Low	**Low**
Kroenke, 2005	Low	Low	Moderate	Moderate	Low	Low	**Low**
Kroenke, 2013	Low	Low	Moderate	Low	Moderate	Low	**Low**
Kwan, 2009	Low	Low	Moderate	Moderate	Moderate	Low	**Moderate**
Mc Cullough, 2013	Moderate	Low	Moderate	Low	Moderate	Low	**Moderate**
Meyerhardt, 2007	Low	Low	Moderate	Low	Low	Low	**Low**
Nechuta, 2012	Low	Low	Moderate	Low	Moderate	Low	**Low**
Petterson, 2012	Moderate	Low	Moderate	Low	Low	Low	**Low**
Ratjen, 2017	Moderate	Low	Moderate	Low	Moderate	Low	**Moderate**
Richman, 2010	Moderate	Low	Moderate	Low	Moderate	Low	**Moderate**
Richman, 2012	Moderate	Low	Moderate	Low	Moderate	Low	**Moderate**
Richman, 2013	Low	Low	Moderate	Low	Moderate	Low	**Low**
Shu, 2009	Moderate	Low	Moderate	Low	Low	Moderate	**Moderate**
Song, 2013	Moderate	Low	Moderate	Low	Moderate	Low	**Moderate**
Song, 2018	Low	Low	Moderate	Low	Low	Low	**Low**
Tamakoshi, 2017	Moderate	Low	Moderate	Low	Moderate	Low	**Moderate**
Van Blarigan, 2015 (colorectal)	Low	Low	Moderate	Low	Moderate	Low	**Low**
Van Blarigan, 2015 (prostate)	Moderate	Low	Moderate	Low	Moderate	Low	**Moderate**
Yang, 2014	Moderate	Low	Moderate	Low	Moderate	Low	**Moderate**
